# Persistent Coagulase-Negative Staphylococcal Bacteremia in Neonates: Clinical, Microbiological Characteristics and Changes within a Decade

**DOI:** 10.3390/antibiotics11060765

**Published:** 2022-06-02

**Authors:** Venetia Bellou, Despoina Gkentzi, Nikolaos Giormezis, Aggeliki Vervenioti, Iris Spiliopoulou, Gabriel Dimitriou

**Affiliations:** 1 Department of Paediatrics, Medical School, University of Patras, Rion, 26504 Patras, Greece; ven_bellou@windowslive.com (V.B.); aggelikivervenioti@upatras.gr (A.V.); gdim@upatras.gr (G.D.); 2 Department of Microbiology, Medical School, University of Patras, Rion, 26504 Patras, Greece; ngiormezis@upatras.gr (N.G.); spiliopl@upatras.gr (I.S.)

**Keywords:** *Staphylococcus*, *CoNS* bacteremia, NICU, persistent, biofilm, *ica* operon

## Abstract

Atypical outbreaks of persistent *coagulase-negative staphylococci (CoNS)* bacteremias, defined as three or more consecutive positive blood cultures with the same *CoNS* species, at least 48 h apart, have been reported in neonatal intensive-care units (NICUs). Our aim was to describe the profile of these cases in our NICU over a two-year period with the objective of assessing possible changes within a decade. Demographics, clinical and microbiological data were recorded for all *CoNS* bacteremias in our tertiary NICU during 2016–2017 and compared with the results of the same study in 2006–2007. Fifty-six cases of *CoNS* sepsis were recorded. Fourteen (25%) of them were persistent. There were no significant differences in demographic and clinical characteristics between cases with persistent vs. non-persistent bacteremia. *Staphylococcus epidermidis* was the most common species. In logistic regression analysis, biofilm production (*β* = 2.464, *p* = 0.04) was the most significant determinant for the development of persistent *CoNS* bacteremia. Our isolates were less likely to produce biofilm and carry *ica* operon as compared to those of 2006–2007. The cases of persistent *CoNS* sepsis have decreased within a decade, which could be attributed to the implementation of intensive infection control practices. Biofilm production remains the most important risk factor.

## 1. Introduction

*Coagulase-negative staphylococci (CoN*S) are a subgroup of staphylococci, which can be distinguished from *Staphylococcus aureus* and *Staphylococcus intermedius* by their lack of ability to produce coagulase. Coagulase secretion is a key virulence strategy in the pathogenesis and persistence of staphylococcal diseases and has often been used to distinguish *S. aureus* from other staphylococci [1]. *Staphylococci* were historically classified into two groups: one that included *S. aureus*, considered more pathogenic and thus a “major pathogen,” and a second including other staphylococci that were lumped together as “minor pathogens” and termed the *CoNS* [1]. Another classification scheme adopted in more recent literature involves grouping all staphylococci other than *S. aureus* into a single category, *non-aureus staphylococci (NAS*) [1]. In addition, *coagulase-negative* variants of *S. aureus* are known to exist, some of which can have similar pathogenicity to their coagulase-positive variants [1,2]. *CoNS* are originally described as ubiquitous commensals of the healthy human skin and mucosa. *Coagulase-negative staphylococci* species, in particular *Staphylococcus epidermidis*, are among the most important pathogens involved in hospital-associated bloodstream infections and infections related to vascular catheters and prosthetic devices. This is due to the fact that they have higher rates of biofilm production and a higher ability to adjust through genetic exchange [2]. *CoNS* have been identified as the leading cause of late-onset sepsis (LOS) in neonatal intensive-care units (NICUs) [3,4,5,6,7,8]. *Staphylococci* can cause up to 90% of LOS in NICUs [3,4,5,6,7,8,9,10]. *S. aureus* represents around 10% of LOS, whereas the proportion of *CoNS* ranges from 30% to 60% [8,9,11]. *S. epidermidis* is the dominant species of *CoNS*, but other species such as *S. capitis, S. haemolyticus, S. hominis* and *S. warneri* have also been reported as pathogens responsible for systemic neonatal infection. *CoNS*, particularly *S. epidermidis*, are the most common organisms found in mucocutaneous sites and the nasopharynx during the first week of life [12]. Risk factors reported for *CoNS* sepsis include extreme prematurity and very low birth weight, as well as the use of intravascular catheters and prolonged parenteral nutrition [9,10,11]. *CoNS* have been traditionally considered to have low virulence and have not been associated with significant morbidity and mortality [13,14,15].

Atypical outbreaks of persistent *CoNS* bacteremia, despite aggressive antibiotic therapy, have been reported in NICUs [15,16,17,18,19]. The increasing prevalence of *CoNS* infection is attributable to their increasing antibiotic resistance and their ability to produce biofilms [19,20]. The biofilm formation facilitates the pathogenicity of *CoNS* by enhancing their ability to adhere to surfaces of invasive devices such as pacemakers, catheters and prosthetic heart valves, as well as smooth plastic and tissue surfaces [21,22]. This mechanism is vital for the persistence of *CoNS,* as biofilm-producing strains are characterized by greater resistance to antibiotics. The bacterial cells within the biofilm are embedded in an exopolysaccharide matrix, which affords the bacterial population protection from host defense mechanisms and antimicrobial agents [20]. The biofilm of *CoNS* is composed of the layer of an extracellular polymeric substance called polysaccharide intercellular adhesion (PIA) matrix, which is encoded by *ica* operon (*icaA, icaB icaC* and *icaD* genes) [23]. The PIA mediates the intercellular adherence of bacteria and the multilayer accumulation of the biofilm, making them resistant to antimicrobial agents and to the immunological response of the host [23,24,25,26].

In our previous report, persistent *CoNS* sepsis was not related to most of the known clinical risk factors, and it was associated with severe thrombocytopenia. Isolates associated with persistent bacteremia were more likely to produce biofilm, independently of the presence of the *ica* operon [17].

We hypothesized that the implementation of infection control practices and antimicrobial stewardship interventions might have led to changes in the characteristics of persistent *CoNS* bacteremia throughout the years. Therefore, the aim of the present study was to describe the clinical and microbiological profile of cases with persistent *CoNS* bacteremia in our NICU during a two-year period and compare our findings with the ones of the study conducted a decade ago in the same setting with the view of assessing trends over time.

## 2. Results

During the study period (2016–2017), 56 cases of neonates with late-onset *CoNS* sepsis were identified (7.4% of all NICU admissions). Of them, 14 (25%) cases with persistent bacteremia were recorded. None of the neonates died from these bacteremias, either persistent or not. The demographics, clinical and laboratory characteristics of these cases are shown in Table 1. No differences were demonstrated between the two groups in terms of demographics and clinical risk factors. The percentage of cases though with persistent bacteremia in our study was lower as compared to 2006–2007, 25% vs. 40%, respectively (*p* = 0.05). With regards to laboratory indices, the maximum CRP values were higher in the persistent bacteremia group. A comparison with the respective cases during 2006–2007 is shown in Table 2 (for all cases) and Table 3 (cases with persistent vs. non-persistent bacteremia). There were no significant differences in demographic characteristics between the two study periods. Of note though, during 2006–2007, the presence of central indwelling catheters was more frequently recorded as compared to the current study (Table 2 and Table 3).

Out of a total of 167 *CoNS* isolates, *S. epidermidis* predominated in 55.4% of the cases, followed by *S. haemolyticus* (33.9%), *S. hominis* (7.1%), *S. capitis* (1.8%) and *S. lugdunensis* (1.8%). An average of seven positive blood cultures with the same isolate per case were identified in the group with persistent *CoNS* bacteremias vs. 1.6 isolates per case in the group with non-persistent bacteremias. Nearly half of the isolates were either biofilm-positive or *ica* operon-positive (Table 4). The presence of either of them was not associated statistically significantly with the development of persistent bacteremia, although the percentages of isolates that were *ica* or biofilm-positives were higher in the persistent group (Table 4). We compared the molecular characteristics of the *CoNS* isolates (*ica* operon, biofilm production, *mecA* gene presence) between our study period and 2006–2007 (Table 4), and we found that isolates in the present study were less likely to carry *ica* operon or to produce biofilm.

Table 5 shows the results of the binary logistic regression analysis of factors that might predict persistent *CoNS* bacteremia (dependent variable). For the purpose of this analysis, we used as independent variables the following parameters: intubation, presence of central indwelling catheters, severe thrombocytopenia and biofilm production. We used these factors because these parameters were found to be major determinants of persistent *CοNS* bacteremias in 2006–2007 [15] and were widely regarded as being linked to infections difficult to manage [11,13,14,15,16,17]. The analysis showed that severe thrombocytopenia and *CoNS* biofilm production both had a significant effect on the modeling of dependent variable values (*p* = 0.005 and *p* = 0.042, respectively). In particular, based on the corresponding factor value (e3.587 = 36.139), the relative probability of a neonate with severe thrombocytopenia being diagnosed with persistent *CoNS* bacteremia is approximately 36-fold higher than that of a neonate without severe thrombocytopenia, irrespective of other factors. In addition, the relative probability of a *CoNS* isolate with biofilm causing persistent *CoNS* bacteremia is 11-fold higher (1074.0%) than that of a *CoNS* isolate with no biofilm, irrespective of other factors. The variability of the dependent variable, which is, in our case, the persistent *CoNS* bacteremia, can be explained by 63% (R^2^ = 0.630).

## 3. Discussion

In the present study, we found that the number of cases with persistent *CoNS* bacteremia has decreased as compared to a similar study conducted ten years ago in the same NICU. We also found that *CoNS* isolates were less likely to carry *ica* operon and to produce biofilm. In both periods, the most common species was *S. epidermidis,* followed by *S. haemolyticus* and *S. hominis,* which is in accordance with other studies [14,15,16,17,18,19,27]. None of the neonates in our study died, which is in keeping with most published studies and suggests that *CoNS* have relatively low virulence and are not associated with significant morbidity [13,14].

The reduction in the number of cases with persistent bacteremia in our study could be attributed to stricter infection control practices and antimicrobial stewardship interventions that have been gradually implemented throughout the ten-year period. Optimal strategies to prevent late-onset sepsis include well-structured infection control protocols, less invasive interventions, rational, empirical antibiotic use, hand hygiene and early feeding with breast milk [28]. All the above have been introduced gradually worldwide and in our NICU within a decade and might have led to an overall reduction in healthcare-associated infections. Of note, in 2020–2021, we had 93 cases with *CoNS* bacteremia in our NICU (19 cases with persistent bacteremia and 74 cases with non-persistent bacteremia). The percentage of persistent *CoNS* bacteremia during the period 2021–2022 was 20%, which is lower than the percentage of persistent *CoNS* bacteremia during 2016–2017 (25%), as well as 2006–2007 (40%). Unfortunately, we do not have microbiological data for the period 2020–2021, but there is a trend toward less persistent *CoNS* bacteremia within the years.

As for the demographics of our participants, it is of interest to note that there were no differences between those belonging to the persistent and those in the non-persistent groups. Given the fact that very low birth weight and extreme prematurity are important risk factors for *CoNS* sepsis in NICUs, one might expect that these babies must have also been at higher risk for persistent *CoNS* bacteremia. This finding has been documented in other studies in the field [5,6,7,8,9,10,11,14,15,16,17,18,19]. On the other hand, this particular neonatal population is more likely to have prolonged hospital stays and invasive procedures, which could, in turn, facilitate the development of persistent disease. In other words, it cannot be clear which is the actual route of the problem: host susceptibility or environmental factors. The latter might account for the lack of correlation found in our study between weight/gestational age with a persistent form of the disease. In addition, the population studied included a rather small number of extremely premature and very low birth weight babies, and therefore, differences could not be demonstrated.

Although there was a trend for the higher presence of *ica* operon or biofilm production in the group of persistent bacteremia, the presence of either of them was not statistically significantly associated with the development of the persistent form of the disease. This is contrary to the finding of the study conducted ten years ago in the same NICU [17]. Overall, we had fewer isolates producing biofilm or carrying *ica* operon, which might account for the absence of correlation between them and persistent disease. We could also hypothesize though that the population of pathogens has changed throughout the decade and gradually became less virulent. It could be perhaps that the implementation of infection control practices and restrictions on antibiotics use might have led to less pressure on *CoNS*. As a result, the isolated strains appeared less virulent. We acknowledge that the small number in each group might limit the validity of any conclusions. With regards to the role of *ica* operon it is worth mentioning that in previous studies, the presence of *ica* operon was not related to persistent bacteremia [29]. In addition, in the study conducted in our institution ten years ago, no significant difference in the distribution of the *ica* operon between persistent and non-persistent groups was found [17]. These findings reinforce the opinion that several mechanisms besides PIA synthesis are responsible for bacterial adhesion and hence biofilm production [30].

With regards to the biofilm formation and resistance to the antibiotic of *Staphylocci,* the exact mechanism is not yet clear. It is highly probable that multiple factors work together to protect biofilm cells from antibiotic treatment. The matrix plays an important role in antibiotic resistance by limiting antimicrobial agents’ penetration into the biofilm and by providing physical protection to the aggregated biofilm cells [31]. As we know, some bacteria harbor antibiotic-resistant genes on plasmids. In biofilms, the frequency of horizontal plasmid transfer is much higher than between planktonic cells. Moreover, biofilm cells have a small metabolic, growth and division rate. These features make biofilm bacteria insensitive to antibiotic drugs that target dividing cells. In biofilms, there is a small subpopulation of cells called persister cells. These cells act as disease reservoirs that could reactivate into infectious particles once the antibiotic stress has been removed [31].

In the logistic regression analysis, intubation and central indwelling catheters were not found to be independently associated with the risk for persistent disease. This could be explained by the fact that these factors could account for the development of late-onset *CoNS* sepsis but not necessarily for the more aggressive form of the disease. The latter could be triggered by certain *CoNS* virulent factors, such as their ability to produce biofilm [30]. The logistic regression analysis in our cohort highlighted the role of biofilm formation as an independent factor accounting for the development of *CoNS* bacteremia.

The limitations of the study need to be acknowledged. First, it is a retrospective and single-institution study. Second, only the presence of *icaA* and *icaD* genes were measured as markers of *ica* positive genotype, and it could be that the expression of other genes might account for *CoNS* virulence. Third, we did not investigate the role of other proteins that might affect biofilm formation. In addition, we cannot be certain as to whether the decrease in the incidence of persistent disease is due to the improvement of infection control practices or due to the change in the molecular characteristics of the *CoNS per se,* making them less virulent. Finally, in order to assess more accurately trends in incidence, clinical and microbiological profile of persistent *CoNS* bacteremia, we ideally need an annual recording of cases for a longer period.

## 4. Materials and Methods

Neonates with late-onset *CoNS* bacteremia were studied over a 2-year period, 2016–2017, and compared to data from 2006–2007 [17]. This study was performed in the NICU of the University General Hospital of Patras, Greece, a level III referral unit, from January 2016 to December 2017. The study was approved by the Ethics Committee of the University General Hospital of Patras (806/11.12.2018). Informed parental consent was not required as the patient data were analyzed anonymously.

All neonates diagnosed with late-onset *CoNS* sepsis, defined as at least one positive blood culture drawn after 72 h of age in the presence of signs of infection, were identified, and those with persistent *CoNS* bacteremia were used for data extraction. Persistent *CoNS* bacteremia was defined as three or more consecutive positive blood cultures with the same *CoNS* species, at least 48 h apart, during a single septic episode. Neonates with two blood cultures with different susceptibilities were excluded from the study. All blood samples for culture were obtained by peripheral venipunctures with a sterile technique after appropriate skin disinfection. Demographical, clinical and microbiological variables at the onset of the septic episode (defined as the time of the first obtained positive blood culture) were reviewed from our medical records.

Demographic data, including sex, gestational age, birth weight and postnatal age, were collected from the patients’ chart reviews. The presence of risk factors for *CoNS* sepsis was also reviewed and included: intubation, use of central indwelling catheter and total parenteral nutrition (TPN) administration.

The clinical profile of neonates was evaluated on the basis of clinical manifestations at the onset of the septic episode, which included temperature instability (rectal temperature of <36.5 °C or >37.5 °C), respiratory deterioration (increased number of oxygen desaturations, increase in oxygen requirement, need for ventilatory support and increased number of apneas), circulatory deterioration (hypotension and signs of peripheral hypoperfusion) and feeding intolerance (abdominal distension or significant aspiration of gastric residue, that required a decrease of >20% or cessation of feeding for at least 24 h). The minimum platelet counts during the septic episode (a platelet count of <80 × 10^9^/L was defined as thrombocytopenia, whereas a platelet count of <30 × 10^9^/L was defined as severe thrombocytopenia), the C-reactive protein (CRP) values at the onset of sepsis, maximum CRP levels during the septic episode and the time from the onset of sepsis to the maximum CRP levels were evaluated.

Blood cultures were processed in the microbiology laboratory of our institution according to regular practice into BacT/ALERT PF pediatric FAN vials of the BacT/ALERT 3D system for aerobic and anaerobic bacteria (bioMerieux SA, Marcy-l’Etoile, France). *CoNS* were identified on the basis of colony morphology, Gram stain, and positive catalase and negative coagulase test results (Slidex Staph Plus; bioMerieux, Marcy-l’Etoile, France). Species identification was performed with the VITEK 2 System (bioMerieux, Marcy-l’Etoile, France). When phenotypic identification showed efficacy lower than 99%, a molecular method based on the tuf gene was applied [32]. Biofilm formation was tested by the quantitative microtitre plate assay using the reference *S. epidermidis* ATCC35984 (RP62A), slime producing/ica-positive strain, and ATCC12228, slime-negative/ica-negative strain, as positive and negative controls, respectively [33]. Amplification of two genes of the *ica* operon (*icaA* and *icaD*) as well as the *mecA* genes (encoding penicillin-binding protein 2a, conferring resistance to methicillin) was performed by PCRs with specific primers, according to previously described protocols [34,35].

Categorical variables are expressed as absolute and relative frequencies, while continuous variables are presented as means ± standard deviations. The chi-square test was used for comparisons between categorical variables with parametric data distribution, whereas the Mann–Whitney U-test was used for comparisons of not normally distributed data. For continuous variables, normally distributed t-test was used. To assess the effect of various factors on predicting the risk of persistent *CoNS* bacteremia, binary logistic regression analysis was used. The regression analysis results are expressed as the coefficients of model (*β*) and their standard errors (SE). Statistics were performed using the SPSS v.17 software (SPSS, Chicago, IL, USA). The level of significance was set to 0.05 for all analyses.

## 5. Conclusions

In conclusion, in the present study, persistent *CoNS* sepsis cases in our NICU have declined within a decade, which could be attributed to the implementation of more structured and intensive infection control practices. This persistent pattern of sepsis was found to be related to the most known molecular characteristics previously described, with biofilm production being the most important. To our knowledge, similar studies have not addressed this issue so far. Further studies are required to assess trends and differences in the patterns of persistent *CοNS* bacteremia over time and to assess the impact of antimicrobial stewardship interventions and preventative measures of hospital-acquired infections.

## Figures and Tables

**Table 1:** Demographics, clinical and laboratory characteristics of the studied population (*n* = 56), cases with persistent (*n* = 14) and cases with non-persistent (*n* = 42) *CoNS* bacteremia the period 2016–2017.

Demographics	All Cases *n* = 56	Persistent *n* = 14	Non-Persistent *n* = 42	*p* Value
Gender				
Male	28	5	23	0.217 ^a^
Female	28	9	19	
Gestational age (weeks) *	33.6 (3.7)	34.4 (3.2)	33.3 (3.9)	0.403 ^b^
Birth weight (g) *	2102 (973)	2142 (823)	2090 (1025)	0.887 ^b^
Clinical practice risk factors				
Intubation	15	3	12	0.601 ^a^
Central indwelling catheters	5	2	3	0.231 ^a^
Parenteral nutrition	24	6	18	0.877 ^a^
Clinical characteristics				
Temperature instability	28	9	19	0.272 ^b^
Food intolerance	31	8	23	0.877 ^a^
Respiratory deterioration	16	4	12	0.601 ^a^
Circulatory deterioration	7	4	3	0.036 ^a^
Laboratory indices				
Thrombocytopenia (<80 × 10^9^/L)	7	3	4	0.243 ^a^
Severe thrombocytopenia (<30 × 10^9^/L)	2	1	1	0.406 ^a^
Maximum CRP (mg/dL) *	7.1(5.4)	12 (4.7)	5.4 (4.8)	0.000 ^b^
Days up to maximum CRP *	1.96(0.9)	2.71 (1.3)	1.71 (0.7)	0.001 ^b^

^a^: χ^2^; ^b^: *t*-test. * Data are presented as mean and SD into brackets.

**Table 2:** Comparison of characteristics among neonates with *CoNS* bacteremias between the periods 2006–2007 (*n* = 72) and 2016–2017 (*n* = 56).

Demographics	2006–2007 *n* = 72	2016–2017 *n* = 56	*p* Value
Gender			
Male	41	28	0.589 ^a^
Female	31	28	
Gestational age (weeks) *	32.6 (3.8)	33.6 (3.7)	0.164 ^b^
Birth weight (g) *	2124 (735)	2102 (973)	0.888 ^b^
Clinical practice risk factors			
Intubation	28	15	0.111 ^a^
Central indwelling catheters	33	5	0.000 ^a^
Parenteral nutrition	41	24	0.114 ^a^
Clinical characteristics			
Temperature instability	52	28	0.038 ^a^
Food intolerance	40	31	0.982 ^a^
Respiratory deterioration	30	16	0.144 ^a^
Circulatory deterioration	41	7	0.000 ^a^
Laboratory indices			
Thrombocytopenia (<80 × 10^9^/L)	37	7	0.000 ^a^
Severe thrombocytopenia (<30 × 10^9^/L)	25	2	0.000 ^a^
Maximum CRP (mg/dL) *	8.1(6.091)	7.1(5.4)	0.330 ^b^
Days up to maximum CRP *	2.16 (1.0)	1.96 (0.9)	0.279 ^b^

^a^: χ^2^; ^b^: *t*-test; * data are presented as mean and SD into brackets.

**Table 3:** Comparison of characteristics among neonates between the periods 2006–2007 and 2016–2017 for the cases with persistent *CoNS* bacteremia and with non-persistent *CoNS* bacteremia.

Demographics	Cases with Persistent *CoNS* Bacteremia		Cases with Non-Persistent *CoNS* Bacteremia	
	2006–2007 *n* = 29	2016–2017 *n* = 14	2006–2007 *n* = 43	2016–2017 *n* = 42
Gender				
Male	16	5	25	23
Female	13	9	18	19
Gestational age (weeks) *	32 (3.8)	34.4 (3.2)	33 (3.7)	33.3 (3.9)
Birth weight (g) *	1940 (736)	2142 (823)	2200 (725)	2090 (1025)
Clinical practice risk factors				
Intubation	16	3 **	12	12
Central indwelling catheters	18	2 ***	15	3 ***
Parenteral nutrition	17	6	24	18
Clinical characteristics				
Temperature instability	24	9	28	19
Food intolerance	19	8	21	23
Respiratory deterioration	14	4	16	12
Circulatory deterioration	19	4 ***	22	3 **
Laboratory indices				
Thrombocytopenia (<80 × 10^9^/L)	27	3 **	10	4
Severe thrombocytopenia (<30 × 10^9^/L)	22	1 **	3	1
Maximum CRP (mg/dL) *	8.9 (6.1)	12 (4.7)	4.0 (3.3)	5.4 (4.8)
Days up to maximum CRP *	2 (0.7)	2.71 (1.3) **	3 (1.0)	1.71 (0.7) **

* Data are presented as mean and SD into brackets. ** *p* < 0.001; *** *p*< 0.05.

**Table 4:** Comparison of molecular characteristics of *CoNS* isolates causing persistent and non-persistent bacteremias throughout the two studied periods.

	Persistent vs. Non-Persistent (2016–2017)		All Cases		Persistent		Non-Persistent	
Study Period	2016–2017	2016–2017	2006–2007	2016–2017	2006–2007	2016–2017	2006–2007	2016–2017
Number of isolates	98	69	170	167	97	98	73	69
*ica*-positive	50	25	129	75 *	74	50 *	55	25 *
Biofilm-positive	50	26	107	76 *	68	50 **	39	26 **
*ica*-positive/biofilm-positive	50	22	91	72	59	50	32	22
*ica*-positive/biofilm-negative	0	3	37	3 *	14	0 *	23	3 *
*ica*-negative/biofilm-negative	43	45	25	88 *	14	43 *	11	45 *
*ica*-negative/biofilm-positive	0	4	16	4 **	9	0 **	7	4
*mecA*-positive	88	69 *	166	157	93	88	73	69

* *p* < 0.001; ** *p* < 0.05.

**Table 5:** Binary logistic regression analysis assessing parameters that might predict the risk of persistent *CoNS* bacteremia the period 2016–2017.

	*β*	Exp (*β*)	SE	Sig.	95% for Exp (*β*)	
					Lower	Upper
Intubation	0.211	1.235	1.143	0.854	0.132	11.591
Central indwelling catheters	1.017	2.766	1.079	0.346	0.334	22.932
Severe thrombocytopenia (<30 × 10^9^)	3.587	36.139	1.264	0.005	3.037	43.083
Biofilm production	2.464	11.747	1.210	0.042	1.095	125.979

## Data Availability

The data presented in this study are available on request from the corresponding author.
